# Supplementary material for: Site-specific metal-support interaction to switch the activity of Ir single atoms for oxygen evolution reaction
Source: Nat Commun. 2024 Jan 16;15:559. doi: 10.1038/s41467-024-44815-0 (PMC10792023; doi:10.1038/s41467-024-44815-0)
Supplement: Supplementary file 1 — Supplementary Information [file 41467_2024_44815_MOESM1_ESM.pdf]

Supplementary Information for

Site-specific metal-support interaction to switch the activity of Ir single atoms for oxygen evolution reaction

Jie Wei[1,7], Hua Tang[1,7], Li Sheng[1,7], Ruyang Wang[4], Minghui Fan[1], Jiale Wan[1], Yuheng Wu[1], Zhirong Zhang[1*], Shiming Zhou[1], and Jie Zeng[1,2,3,5,6*]

[1]Hefei National Research Center for Physical Sciences at the Microscale, University of Science and Technology of China, Hefei, Anhui 230026, P. R. China

[2]Key Laboratory of Surface and Interface Chemistry and Energy Catalysis of Anhui Higher Education Institutes, Department of Chemical Physics, University of Science and Technology of China, Hefei, Anhui 230026, P. R. China

[3]CAS Key Laboratory of Strongly-Coupled Quantum Matter Physics, University of Science and Technology of China, Hefei, Anhui 230026, P. R. China

[4]National Synchrotron Radiation Laboratory, University of Science and Technology of China, Hefei, Anhui 230026, P. R. China

[5]School of Chemistry & Chemical Engineering, Anhui University of Technology, Ma'anshan, Anhui 243002, P. R. China

[6]Institute of Advanced Technology, University of Science and Technology of China, 230031 Hefei, Anhui, P. R. China

[7]These authors contributed equally to this work.

[*]Corresponding author. E-mail: zzhirong@ustc.edu.cn (Z.Z.); zengj@ustc.edu.cn (J.Z.)

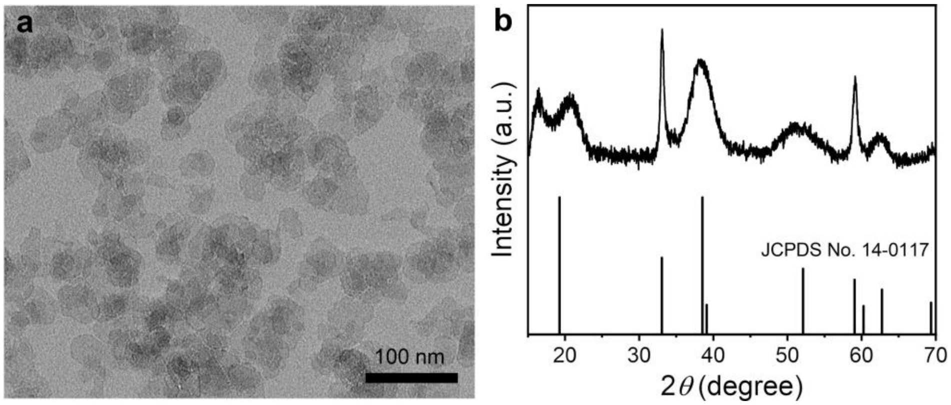

Supplementary Figure 1. Morphological and structural characterizations of Ni LDH nanosheets. (**a**) TEM image and (**b**) XRD pattern of Ni LDH nanosheets. The (001) splitting at 2θ = 19.26^{o} is attributed to the presence of formamide between a fraction of layers in the material[1].

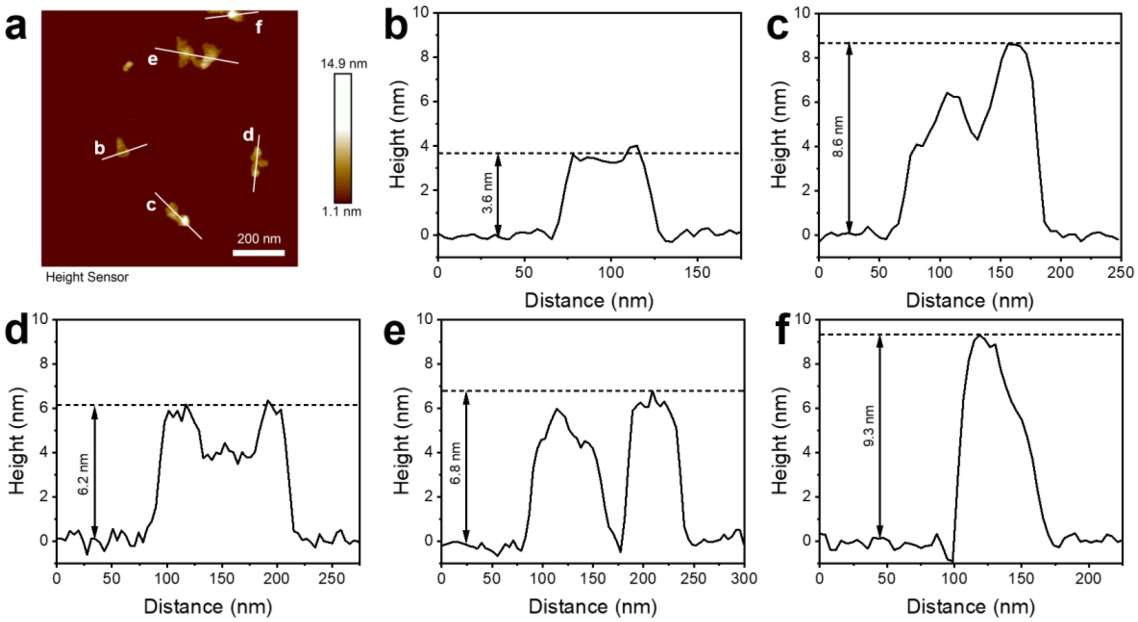

Supplementary Figure 2. AFM characterizations of Ni LDH nanosheets. AFM image (**a**) and corresponding height profiles of Ni LDH nanosheets (**b-f**).

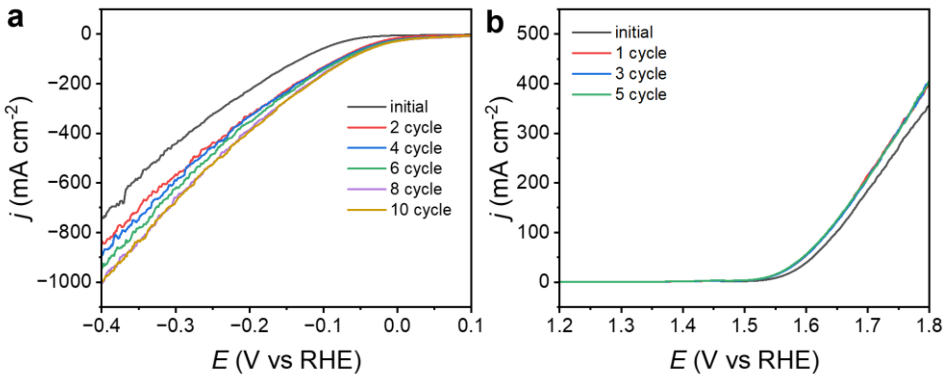

Supplementary Figure 3. The electrochemical deposition curves of (**a**) Ir_1/Ni LDH-T and (**b**) Ir_1/Ni LDH-V with 100 μM $IrCl_4$.

Note:

In the electrochemical cathodic deposition, the current gradually increased with the increase of deposition cycle after the introduction of $IrCl_4$ as the precursor. The increased cathodic current was derived from the activity of Ir single atoms towards hydrogen evolution, suggesting the successful anchoring of Ir atoms on Ni LDH support. In the case of electrochemical anodic deposition, the slight increase of the current after 5 deposition cycles was consistent with the limited improvement in the OER activity of Ir_1/Ni LDH-V compared with Ni LDH.

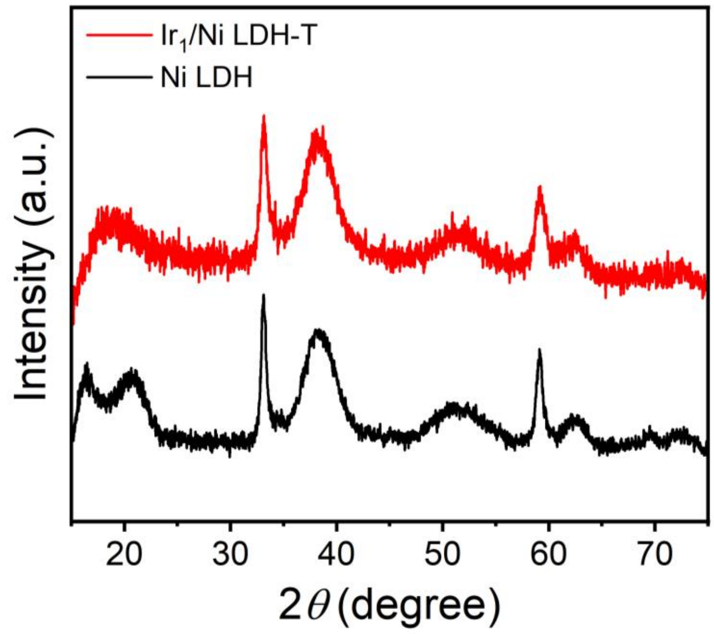

Supplementary Figure 4. XRD pattern of Ir_1/Ni LDH-T.

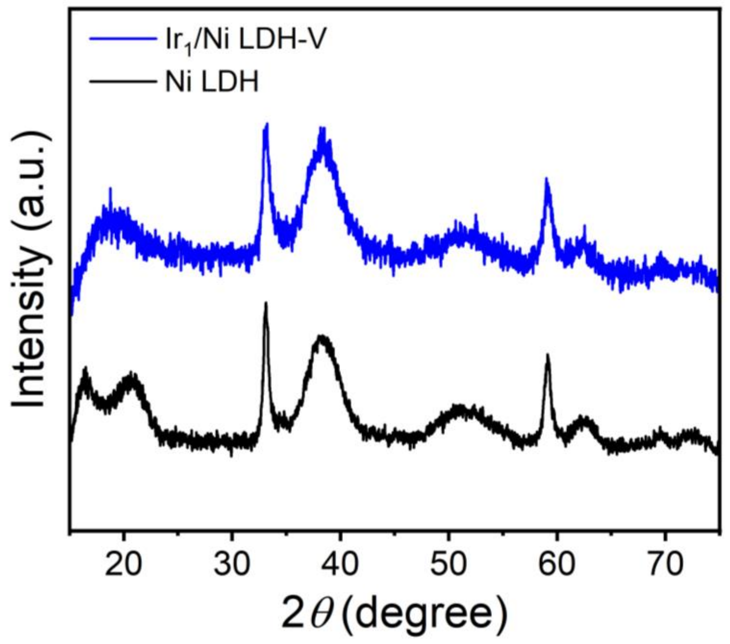

Supplementary Figure 5. XRD pattern of Ir_1/Ni LDH-V.

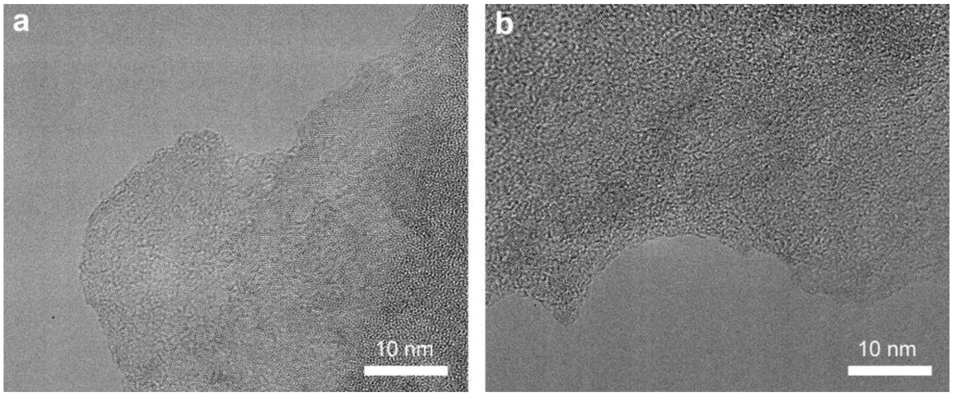

Supplementary Figure 6. HR-TEM of (**a**) Ir_1/Ni LDH-T and (**b**) Ir_1/Ni LDH-V.

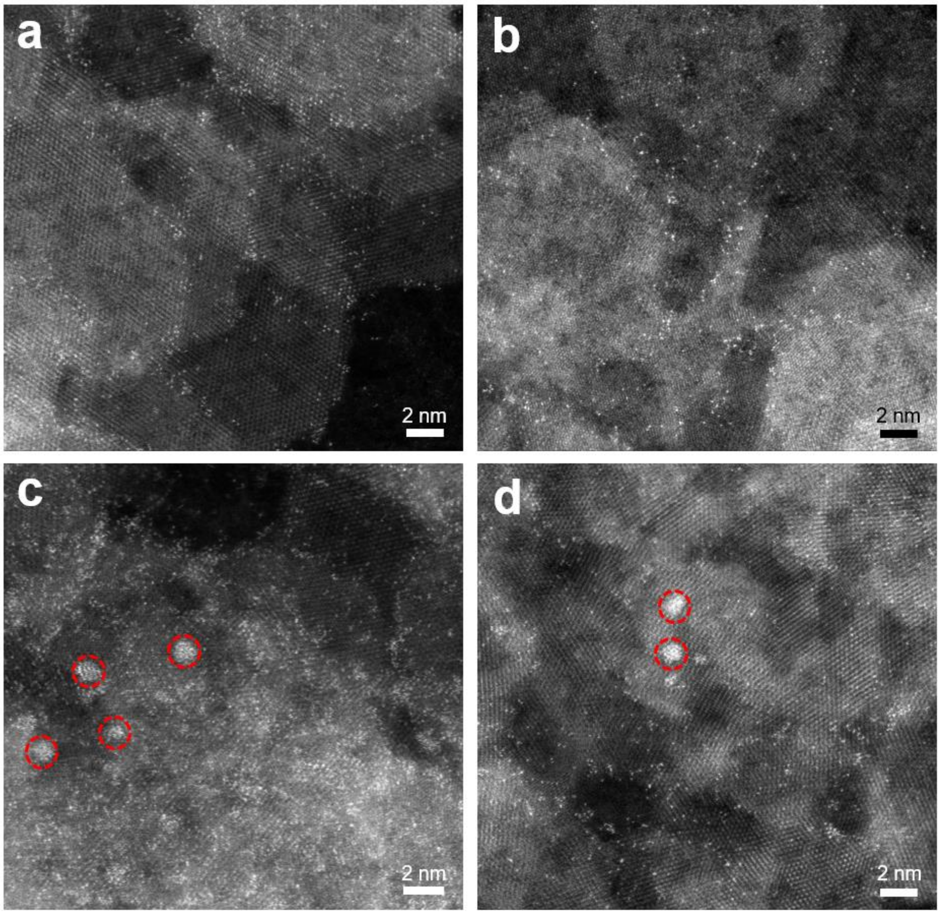

Supplementary Figure 7. HAADF-STEM images of Ir_1/Ni LDH-T and Ir_1/Ni LDH-V obtained at a certain concentration. a, b HAADF-STEM images of Ir_1/Ni LDH-T (**a**) and Ir_1/Ni LDH-V (**b**) obtained at 50 μM $IrCl_4$. **c, d** HAADF-STEM images of Ir_1/Ni LDH-T (**c**) and Ir_1/Ni LDH-V (**d**) obtained at 200 μM $IrCl_4$. Ir single atoms aggregated into clusters when the concentration of $IrCl_4$ was increased to 200 μM.

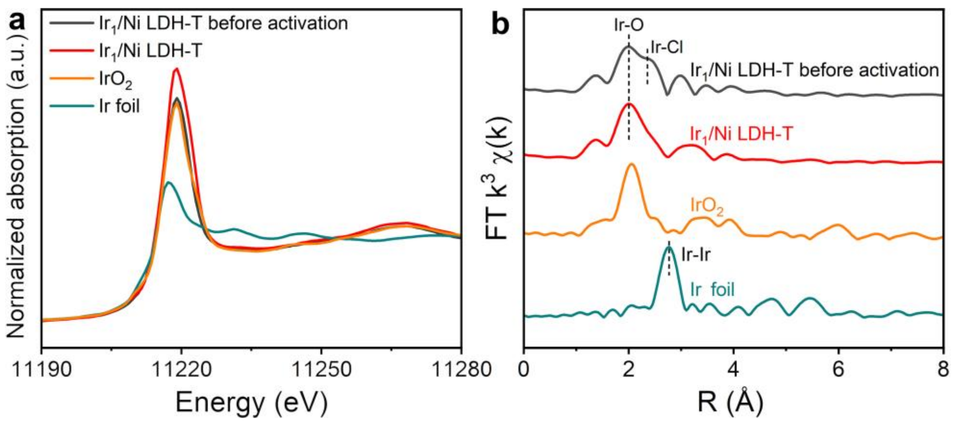

Supplementary Figure 8. XAFS spectra of Ir_1/Ni LDH-T before and after activation. a, b Normalized Ir L_3-edge XANES (a) and EXAFS (b) spectra of Ir_1/Ni LDH-T before and after activation. Ir foil and IrO_2 were used as references. The fitting EXAFS results show that Ir_1/Ni LDH-T exhibits a weak peak at 2.31 Å, which can be attributed to the Ir-Cl bond. The coordination number of Ir-O and Ir-Cl is fitted to be 5.6 and 0.4, respectively. In comparison, we tested XAFS of Ir_1/Ni LDH-T before electrochemical activation. The results showed that the number of coordinates of Ir-O and Ir-Cl was 4.4 and 1.4, respectively (Supplementary Table 2). In this case, the Ir-Cl bond was transformed to Ir-O bond after the OER activation process.

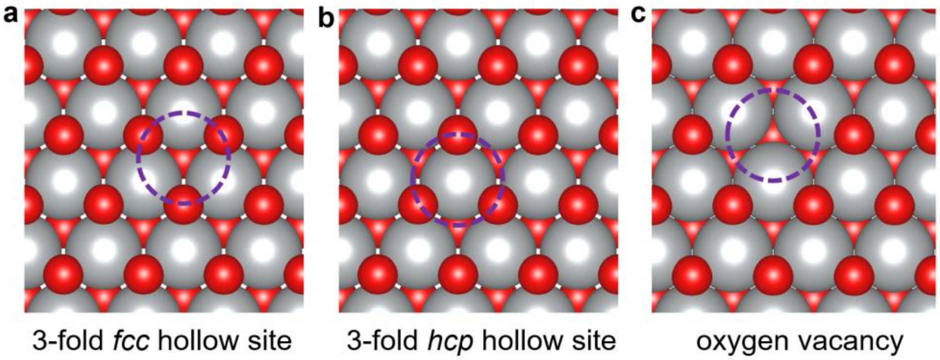

Supplementary Figure 9. Potential anchoring sites of Ni LDH. Three-fold (3-fold) *fcc* hollow site (**a**), 3-fold *hcp* hollow site (**b**), and oxygen vacancy site (**c**). The red and gray represent O and Ni atoms, respectively.

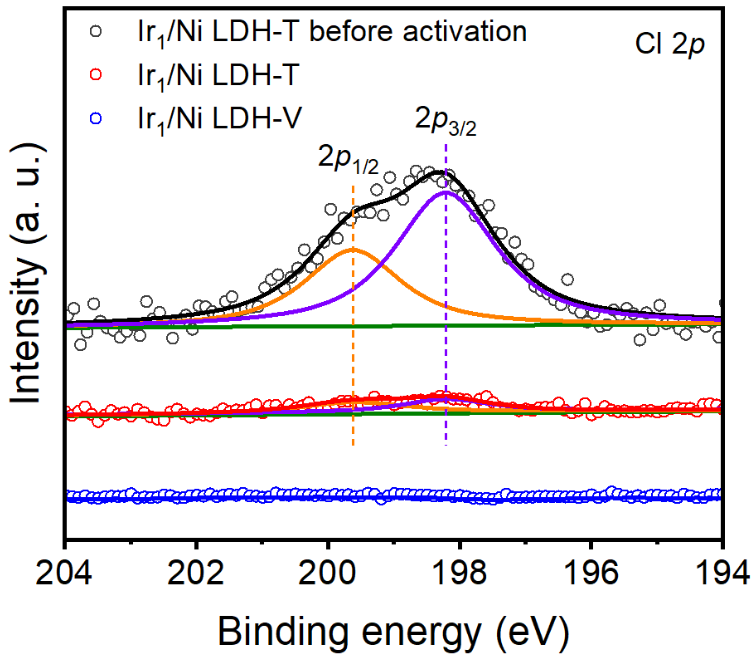

Supplementary Figure 10. Cl 2*p* XPS spectra of Ir_1/Ni LDH-T before activation, Ir_1/Ni LDH-T, and Ir_1/Ni LDH-T.

Note:

The Cl 2*p* XPS spectrum of Ir_1/Ni LDH-T before activation exhibited the peaks of Cl $2p_{1/2}$ and $2p_{3/2}$ at 199.6 and 198.2 eV, respectively. The presence of Cl in Ir_1/Ni LDH-T before activation was attributed to the deposition species originated from the $IrCl_4$ precursor. After the OER activation, the peak intensity of Cl in Ir_1/Ni LDH-T significantly decreased, indicating the oxidative dechlorination during the activation process. In addition, the absence of Cl peak for Ir_1/Ni LDH-V was consistent with the selective deposition of $Ir(OH)_6^{2-}$ after anodic electrochemical deposition.

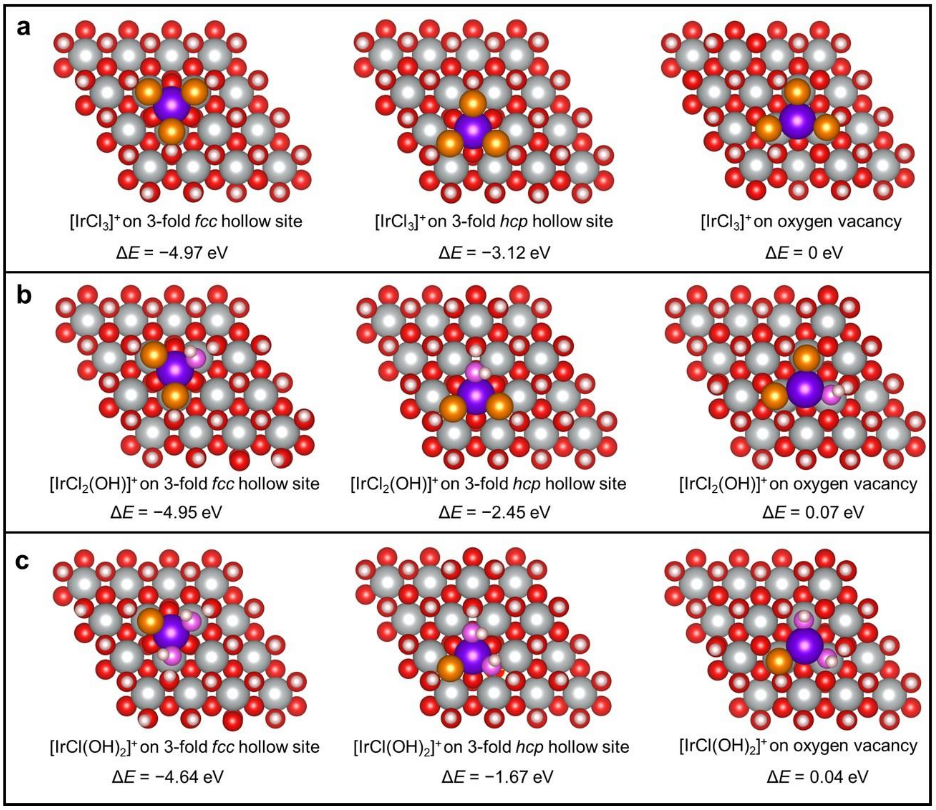

Supplementary Figure 11. Formation energies (ΔE) of $[IrCl_3]^+$ (a), $[IrCl_2(OH)]^+$ (b), and $[IrCl(OH)_2]^+$ (c) anchoring on 3-fold *fcc*, 3-fold *hcp* hollow site, and oxygen vacancy site of Ni LDH (001), respectively. The pink, orange, gray, and purple spheres represent H, Cl, Ni, and Ir atoms, respectively. The red and magenta spheres represent the O atoms bonded to Ni and Ir, respectively.

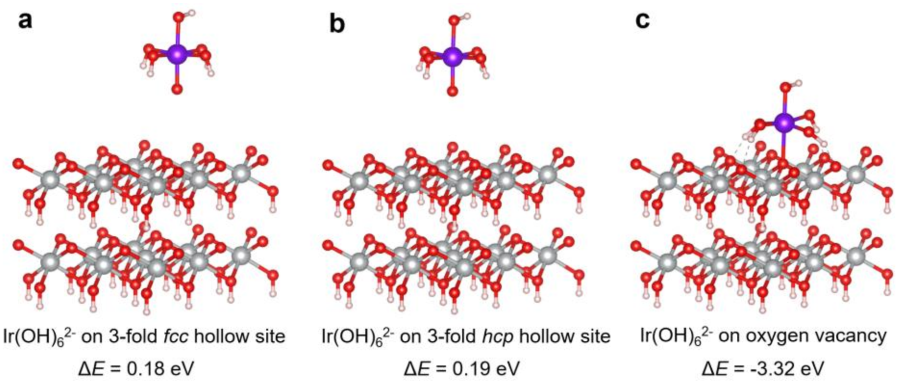

Supplementary Figure 12. Formation energies (Δ*E*) of Ir(OH)$_6^{2-}$ anchoring on three-fold *fcc* (a), three-fold *hcp* hollow site (b), and oxygen vacancy site (c) of NiOOH (001). The pink, red, gray, and purple spheres represent H, O, Ni, and Ir atoms, respectively.

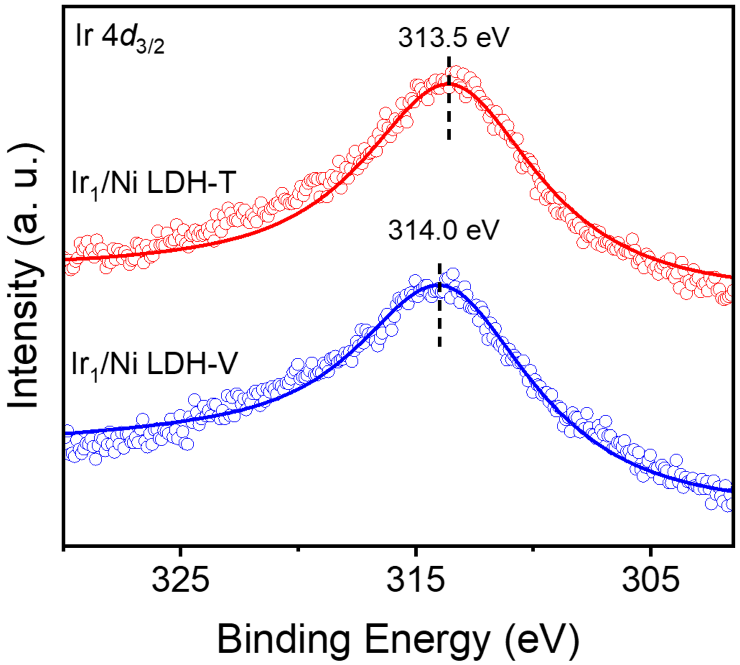

Supplementary Figure 13. Ir 4*d* XPS spectra scanned for 30 times of Ir_1/Ni LDH-T and Ir_1/Ni LDH-V.

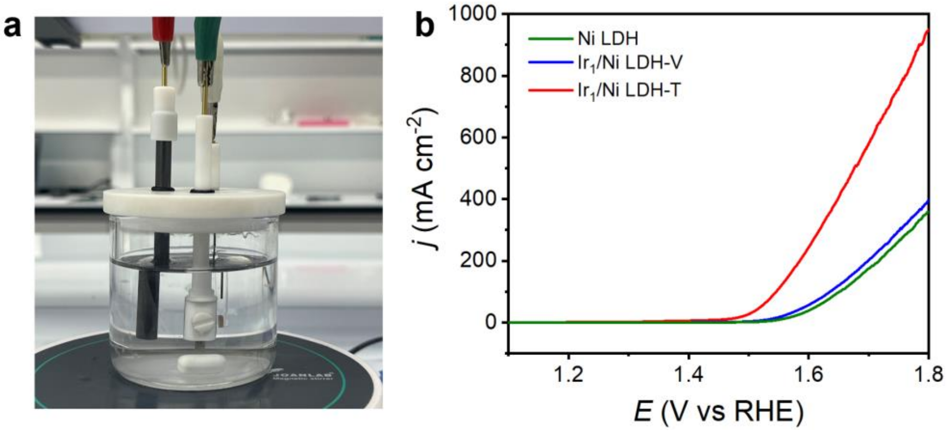

Supplementary Figure 14. (**a**) A photograph of the electrochemical cell used in this work. (**b**) Non-iR corrected polarization curves of Ni LDH, Ir_1/Ni LDH-V, and Ir_1/Ni LDH-T in 1.0 M KOH. The area of working electrode was 0.3×0.3 cm^2 and the R_u value was measured to be ~3.0 Ω.

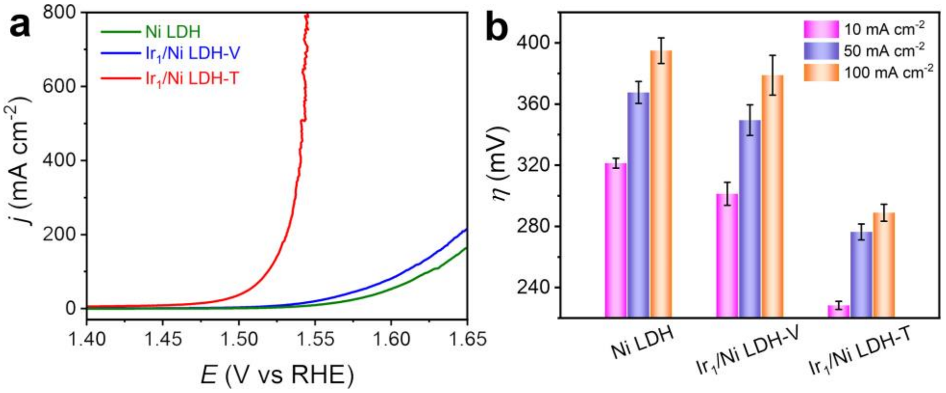

Supplementary Figure 15. The catalytic performance normalized by geometric area of Ir_1/Ni LDH-T, Ir_1/Ni LDH-V, and Ni LDH. (a) Polarization curves of Ni LDH, Ir1/Ni LDH-V, and Ir_1/Ni LDH-T in 1.0 M KOH. (b) Overpotentials of different catalysts at current densities of 10, 50, and 100 mA cm^{-2}, respectively. The error bars correspond to the standard deviation of three independent measurements.

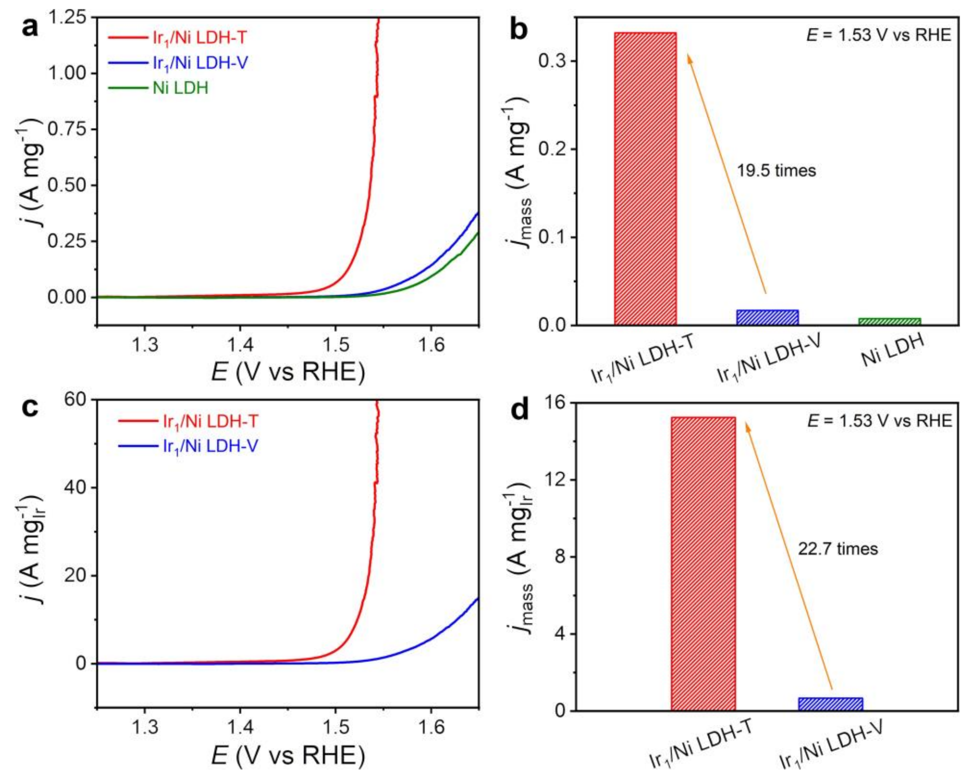

Supplementary Figure 16. Mass activities of Ir_1/Ni LDH-T, Ir_1/Ni LDH-V, and Ni LDH normalizing to different components. (**a**) Polarization curves and (**b**) mass activities normalized by the overall mass of catalysts at an overpotential of 300 mV. (**c**) Polarization curves and (**d**) mass activities at an overpotential of 300 mV normalized by the content of Ir loadings.

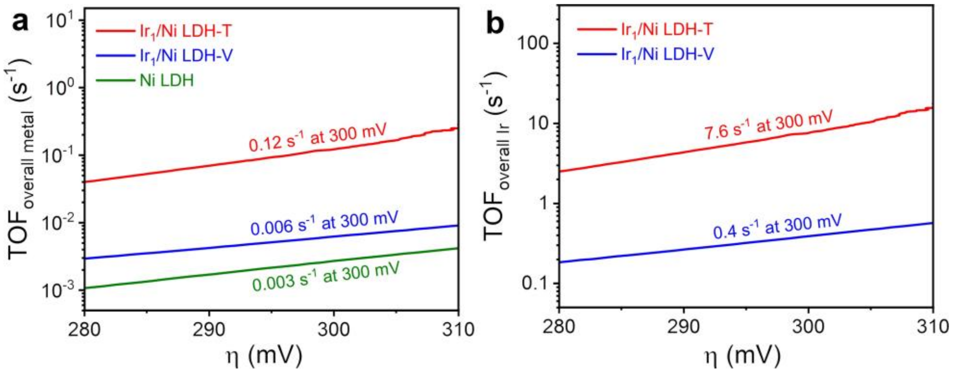

Supplementary Figure 17. TOFs normalized by different components at different overpotentials (η). (**a**) TOFs of Ir_1/Ni LDH-T, Ir_1/Ni LDH-V, and Ni LDH normalized by the number of overall metals. (**b**) TOFs of Ir_1/Ni LDH-T and Ir_1/Ni LDH-V normalized by the number of overall Ir.

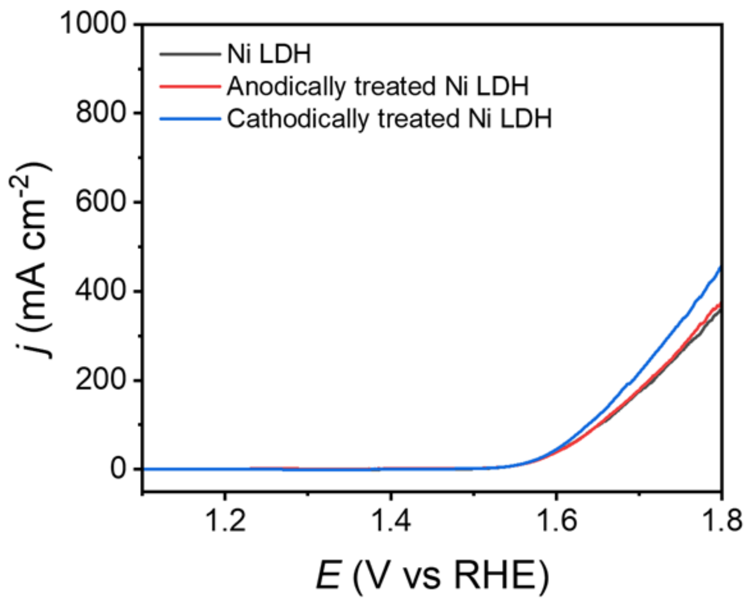

Supplementary Figure 18. Polarization curves of Ni LDH, cathodically treated Ni LDH, and anodically treated Ni LDH.

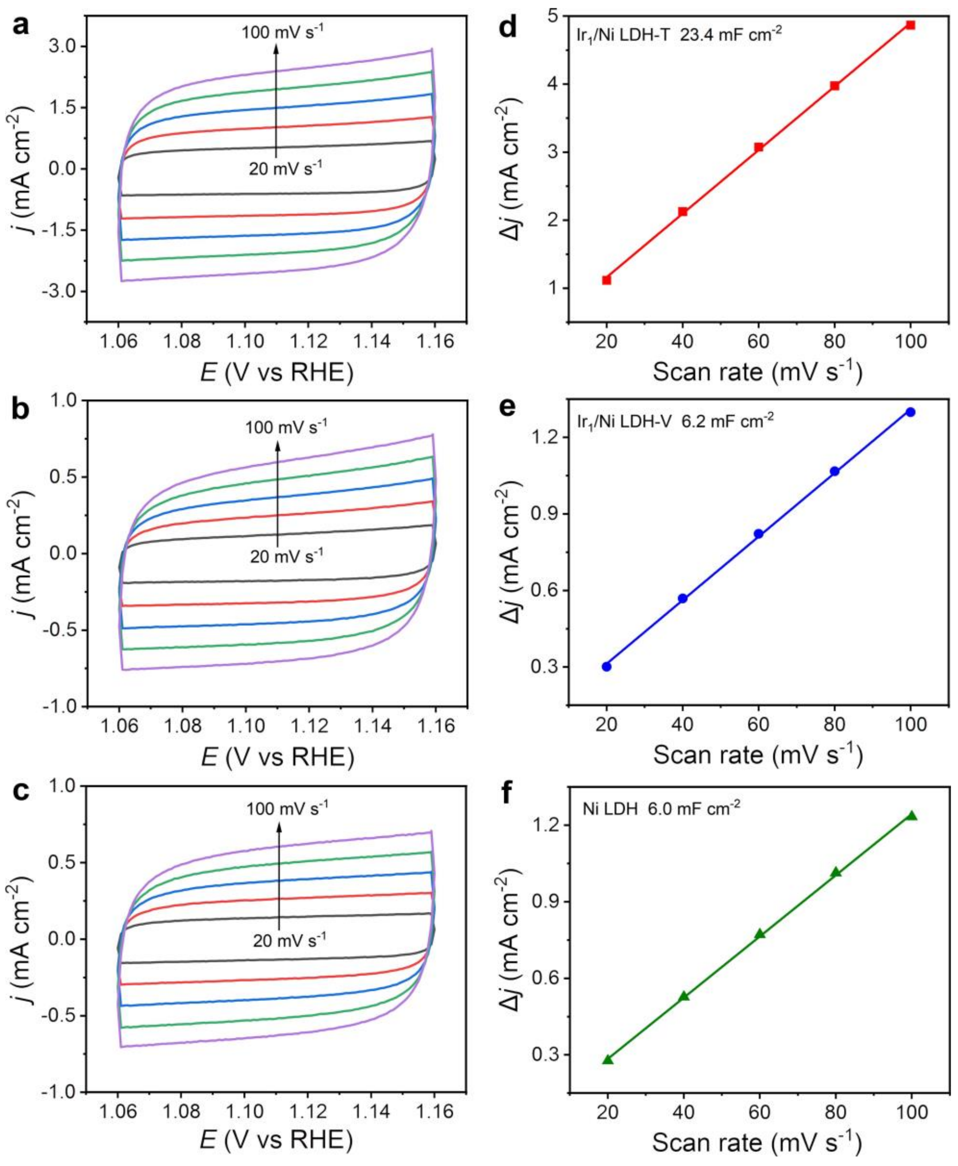

Supplementary Figure 19. CV curves and charging current density differences of Ir_1/Ni LDH-T, Ir_1/Ni LDH-V, and Ni LDH. CVs for (**a**) Ir_1/Ni LDH-T, (**b**) Ir_1/Ni LDH-V, and (**c**) Ni LDH catalysts at different scan rates from 20 to 100 mV s^{-1}, respectively. Charging current density differences of (**d**) Ir_1/Ni LDH-T, (**e**) Ir_1/Ni LDH-V, and (**f**) Ni LDH, respectively.

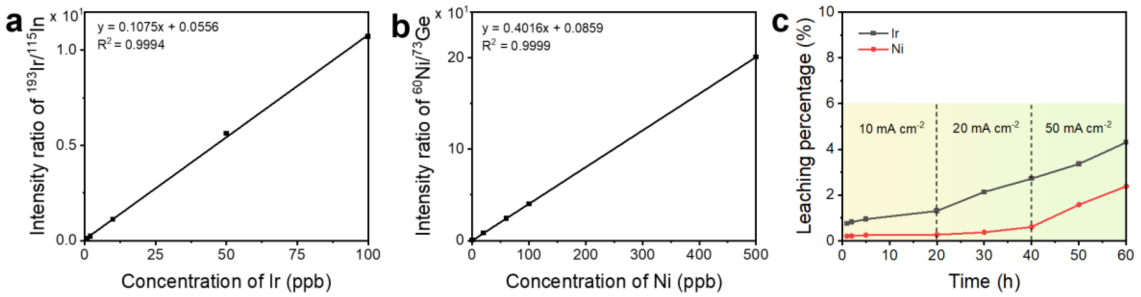

Supplementary Figure 20. The leaching percentage of Ir and Ni after the stability test. Standard curve for the quantification analysis of Ir (**a**) and Ni (**b**) by ICP-MS. The signal ratios of Ir/In and Ni/Ge were plotted on the y-axis against the concentrations of Ir and Ni on the x-axis, respectively. 40 ppb of In and Ge were employed as internal standards for Ir and Ni quantification, respectively. **c** The percentages of leached Ir and Ni detected by ICP-MS after the stability test at 10, 20, and 50 mA cm^{-2} for 20 h, respectively. Leached percentage = (leached mass of Ir or Ni)/(starting mass of Ir or Ni) × 100%.

Note:

2.2 mg of Ni LDH was coated onto a 2 × 2 cm^2 nickel foam, and the electrochemical deposition was carried out under the same conditions as that of Ir_1/Ni LDH-T. The prepared electrodes were tested in a fresh 1.0 M KOH solution. The current density of 10, 20, and 50 mA cm^{-2} for 20 h in 100 mL of 1.0 M KOH electrolytes was applied to test the stability of Ir_1/Ni LDH-T. Before the quantification using ICP-MS, HCl was added into the electrolyte for acidification.

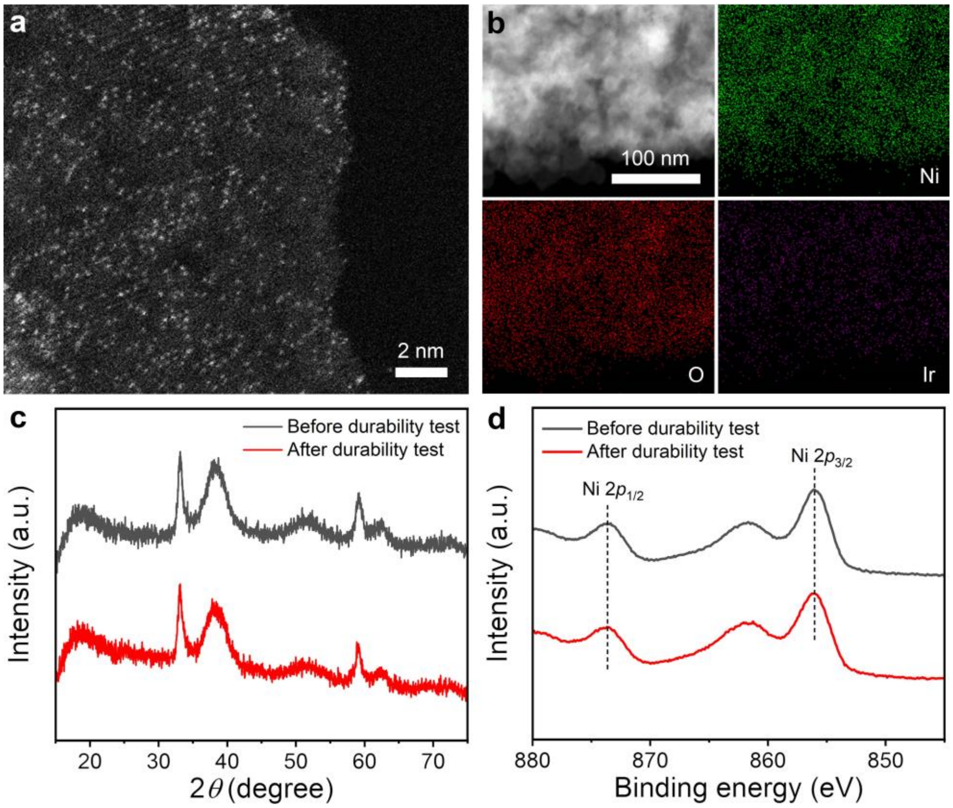

Supplementary Figure 21. Morphological and structural characterizations of Ir_1/Ni LDH-T after durability test. (**a**) HAADF-STEM image, (**b**) EDS elemental mapping, (**c**) XRD pattern, and (**d**) Ni 2*p* XPS spectrum of Ir_1/Ni LDH-T after durability test. XRD pattern and Ni 2*p* XPS spectrum of Ir_1/Ni LDH-T before the durability test were used as references.

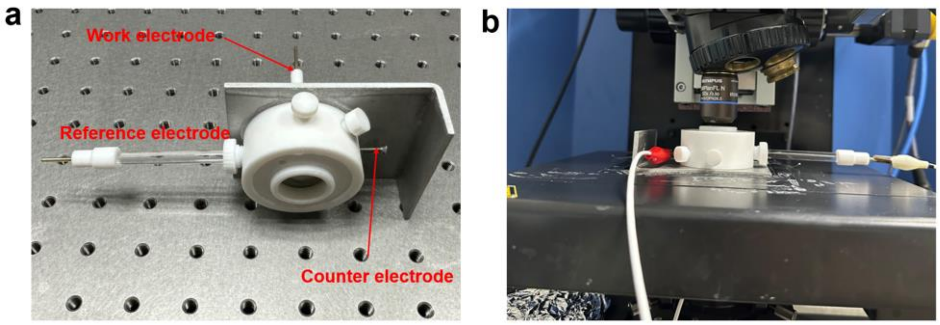

Supplementary Figure 22. Photographs of the homemade in situ Raman cell (a) and the Raman cell during the operation (b).

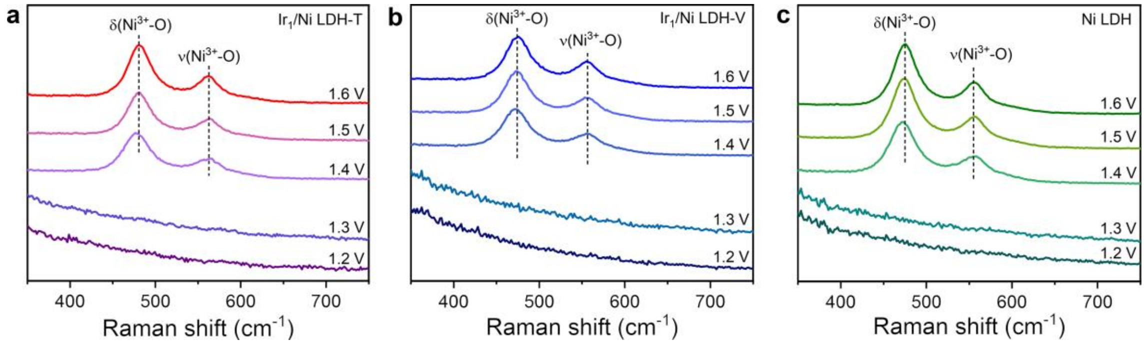

Supplementary Figure 23. In situ electrochemical Raman spectra of (a) Ir_1/Ni LDH-T, (b) Ir_1/Ni LDH-V, and (c) Ni LDH in 1.0 M KOH solution.

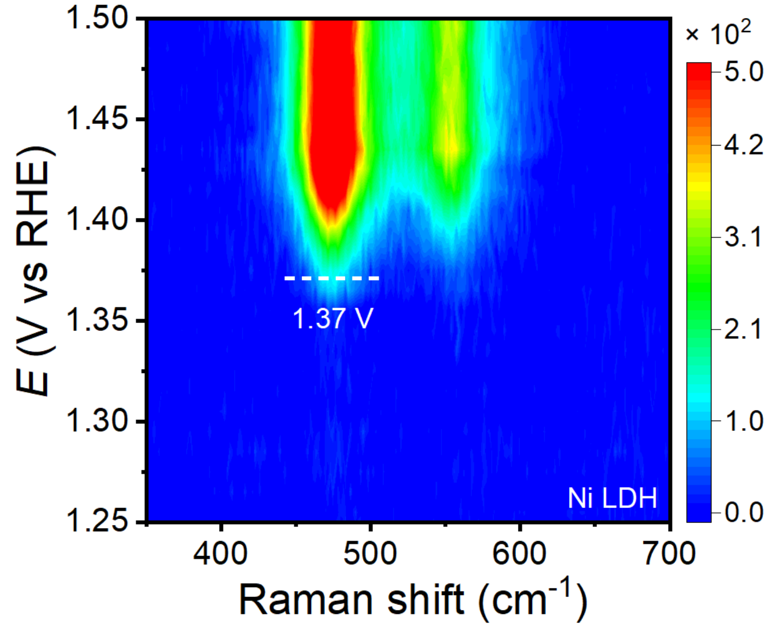

Supplementary Figure 24. In situ Raman spectra of Ni LDH acquired during the linear sweep voltammetry measurement, in which the dash lines mark the required potential for the transition from Ni^{2+} to Ni^{3+}.

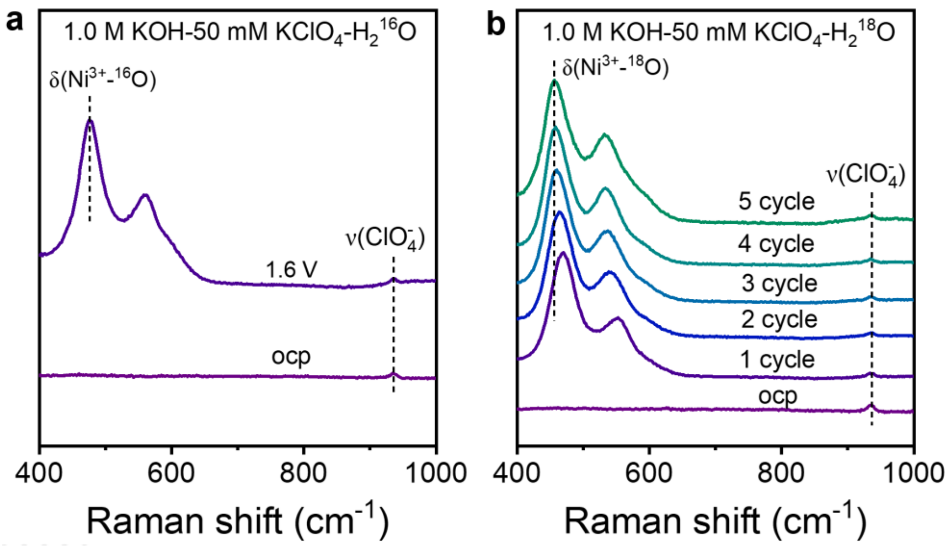

Supplementary Figure 25. In situ Raman test with the addition of ClO_4^- as the internal standard. a In situ Raman spectra of Ni LDH at 1.6 V vs RHE in 1.0 M KOH-$H_2{}^{16}O$ and 50 mM $KClO_4$. **b** Raman spectra of Ni LDH after LSV measurement from 0.50 to 1.65 V vs RHE for five cycles in 1.0 M KOH-$H_2{}^{18}O$ and 50 mM $KClO_4$.

Note:

To confirm the shift of δ(Ni^{3+}-O), we conducted the in-situ Raman test with the addition of 50 mM $KClO_4$ as an internal standard. The peak at 935 cm^{-1} was assigned to the symmetric stretching mode of ClO_4^- (ν(ClO_4^-)) in the 1.0 M KOH-$H_2{}^{16}O$ solution. During LSV measurement from 0.50 to 1.65 V vs RHE for five cycles in 1.0 M KOH-$H_2{}^{18}O$ solution, the peak of ν(ClO_4^-) remained unchanged whereas the peak of δ(Ni^{3+}-O) gradually shifted to 457 cm^{-1}. This result indicated that the oxygen in Ni LDH was successfully labelled with ^{18}O.

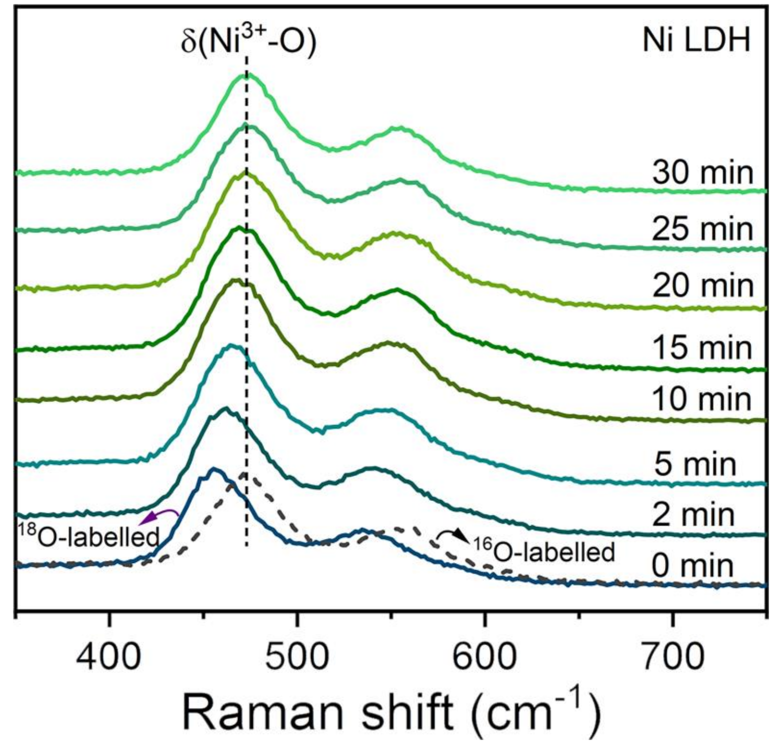

Supplementary Figure 26. Oxygen isotope exchange experiments. In situ Raman spectra of ^{18}O-labelled Ni LDH acquired at 50 mA cm^{-2} in 1.0 M KOH-$H_2{}^{16}O$ solution.

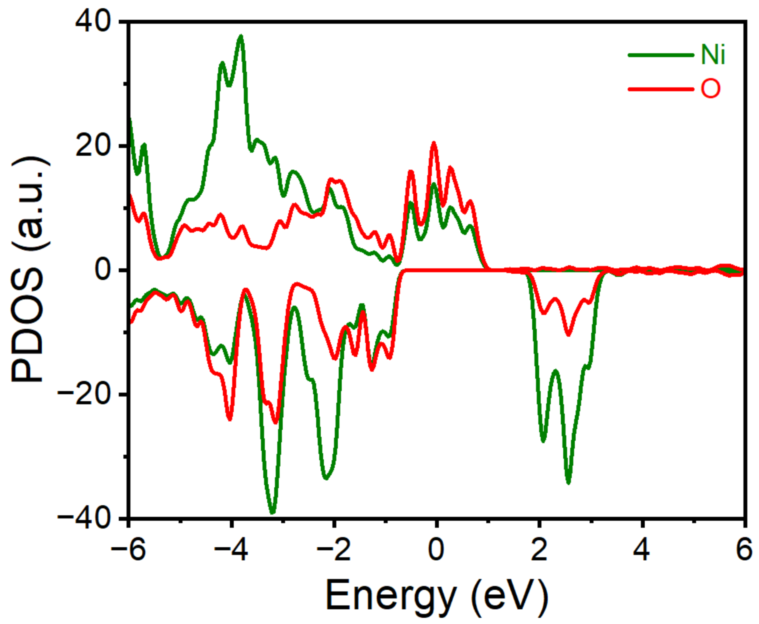

Supplementary Figure 27. **Projected density of states (PDOS) in NiOOH**.

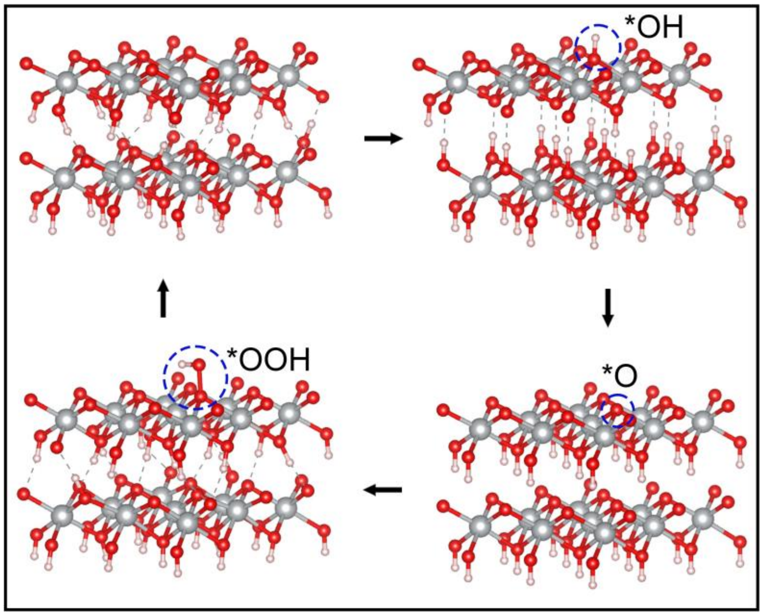

Supplementary Figure 28. The schematic OER pathway for Ni LDH. The pink, red, and gray spheres represent H, O, and Ni atoms, respectively. The reaction intermediates are indicated by blue circles.

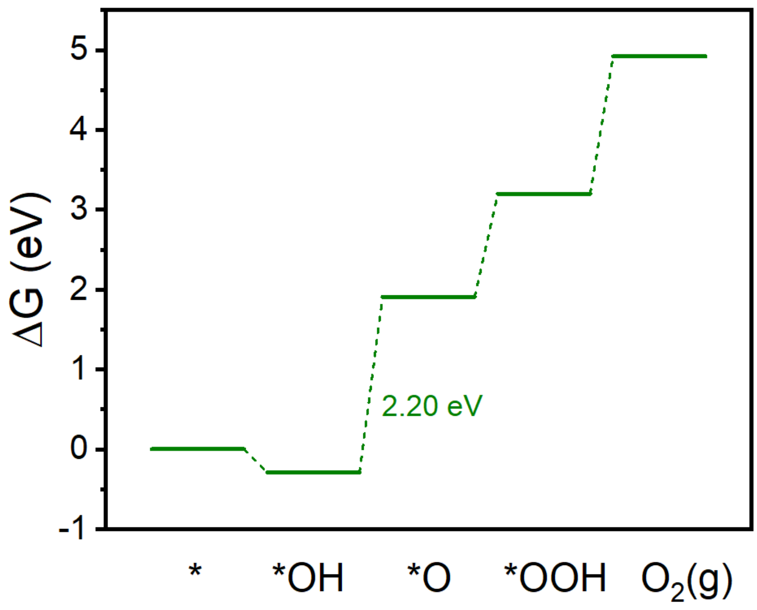

Supplementary Figure 29. Free energy diagrams of Ni sites for NiOOH.

Supplementary Table 1. The loading of Ir in Ir_1/Ni LDH-T and Ir_1/Ni LDH-V with different concentrations of $IrCl_4$.

Concentration of $IrCl_4$	Ir_1/Ni LDH-T	Ir_1/Ni LDH-V
25 μM	0.50 wt%	0.69 wt%
50 μM	1.23 wt%	1.60 wt%
100 μM	2.18 wt%	2.54 wt%
200 μM	2.92 wt%	3.06 wt%

Supplementary Table 2. EXAFS fitting results of Ir_1/Ni LDH-T and Ir_1/Ni LDH-V. The amplitude reduction factor S_0^2 was fixed at the value of 0.82 determined by fitting the data of IrO_2.

Samples	Path	C.N.	R (Å)	$\triangle E_0$	σ^2 (eV)	R factor
Ir_1/Ni LDH-T before activation	Ir-O	4.4	1.98	7.2	0.005	0.11
	Ir-Cl	1.4	2.33	10	0.005	
	Ir-O-Ni	3.0	3.23	18.8	0.015	
Ir_1/Ni LDH-T	Ir-O	5.6	1.98	8.4	0.005	0.012
	Ir-Cl	0.4	2.31	-3.1	0.005	
	Ir-O-Ni	2.8	3.08	11.8	0.003	
Ir_1/Ni LDH-V	Ir-O	6.1	1.99	8.6	0.005	0.001
	Ir-Cl	0	-	-	-	
	Ir-O-Ni	1.4	2.91	2.8	0.014	

Supplementary References

1. Alsaç, E. P. et al. Identification of non-traditional coordination environments for iron ions in nickel hydroxide lattices. *Energy Environ. Sci.* **15**, 2638-2652 (2022).
